# Rosuvastatin exerts cardioprotective effect in lipopolysaccharide-mediated injury of cardiomyocytes in an MG53-dependent manner

**DOI:** 10.1186/s12872-022-02458-3

**Published:** 2022-02-23

**Authors:** Jiawei Zhuang, Gangyi Cheng, Jian Huang, Hongwei Guo, Yiquan Lai, Jiamao Wang, Zhonggui Shan, Shaoyi Zheng

**Affiliations:** 1 Department of Cardiovascular Surgery, Nanfang Hospital, Southern Medical University, Guangzhou, China; 2 Department of Cardiovascular Surgery, The First Affiliated Hospital of Xiamen University, Xiamen, China

**Keywords:** Myocarditis, Rosuvastatin MG53

## Abstract

**Background:**

Myocarditis is a cardiomyopathy associated with the inflammatory response. Rosuvastatin (RS) demonstrates cardioprotective effect in the clinical setting, although its cellular and molecular mechanisms in ameliorating myocarditis are largely unknown. MG53 (muscle-specific E3 ligase Mitsugumin 53), a newly identified striated muscle-specific protein, is involved in skeletal muscle membrane repair. We aimed to explore whether RS mediated the repair of cardiomyocytes in an MG53-dependent manner.

**Methods:**

The RS-induced upregulation of MG53 was determined using RT-qPCR and western blotting. A lipopolysaccharide (LPS)-induced cell inflammatory model was constructed using rat cardiac muscle cell H9C2. Inflammatory injury was evaluated according to the alterations of cell viability, mitochondrial membrane potential, cell apoptosis, and expression of pro-inflammatory cytokines (interleukin-1β, interleukin-6, tumor necrosis factor-α, and monocyte chemoattractant protein-1). Small interfering RNAs (siRNAs) were used to silence MG53. The cardioprotective effect of RS and the inhibition of this protection by MG53 silence were evaluated in the forementioned in vitro model. The underlying mechanism was finally investigated using western blotting to detected the expressions of apoptotic markers (Bcl-2, Bax, Cleaved caspase-9, Cleaved caspase-3), cell cycle regulatory factors (Cyclin A, Cyclin E1, Cyclin D1, CDK2), and components involved in NF-κB signaling pathway (p-IκBa, Iκba, p-p65, p65).

**Results:**

RS ameliorated LPS-induced inflammatory injury. RS upregulated the expression of MG53. MG53 was crucial for the RS-mediated repair response in vitro. Ablation of MG53 inhibited the RS-mediated protective effect. Furthermore, RS and MG53 interact in multiple signaling pathways to modulate recovery.

**Conclusion:**

RS exerts cardioprotective effect in an MG53-dependent manner. MG53 may serve as a novel drug target for myocarditis treatment.

**Supplementary Information:**

The online version contains supplementary material available at 10.1186/s12872-022-02458-3.

## Introduction

Myocarditis, an infectious disease, is commonly caused by Type B Coxsackievirus (CVB) and characterized by myocardial inflammatory cell infiltration and non-ischemic myocytic necrosis. The majority of CVB infections are asymptomatic [1, 2]. However, at least 70% of the world population is estimated to have anti-CVB antibodies. The severe symptoms can vary from a mild infection to sudden cardiac arrest in young and healthy individuals [3]. Recently, it is reported that Coronavirus Disease 2019 can also induce myocarditis [4]. Myocarditis contributes much to the mortality of people less than 40 years old and constitutes approximately 20% of cardiovascular disease (CVD) events. The dilated cardiomyopathy caused by myocarditis can progress to cardiac arrest, whose 10-year survival probability is < 40% [5].

Statins, 3-hydroxy-3-methylglutaryl coenzyme A (HMG-CoA) inhibitors, can suppress the synthesis of cholesterol, which plays a critical role in CVD. Use of statins greatly decrease CVD-related morbidity and mortality [6, 7]. In addition to their lipid-lowering effects, statins exhibit pleiotropic effects. Recently, several studies have indicated that statins may attenuate myocarditis [5]. The predominant effect of statins on myocarditis therapy due to suppression of cross-talk between lymphocytes and antigen presenting cells (APCs) [8]. Statins downregulate the expression of histocompatibility complex class II in APCs, resulting in the depression of Th1 differentiation and activation, which inhibits the release of pro-inflammatory cytokines such as tumor necrosis factor alfa (TNF-α), interleukin 1β, interleukin 6 (IL-6), and interleukin 8 [9]. Rosuvastatin (RS) is one kind of statins. In vivo study has demonstrated that RS exerts anti-inflammatory effects, reducing the expression levels of TNF-α and IL-6 [10]. Moreover, RS significantly reduces myocardial apoptosis [11]. However, the underlying mechanism of RS on myocarditis remains elusive.

The muscle-specific E3 ligase Mitsugumin 53 (MG53, or TRIM72) is most abundantly expressed in the myocardium and its mutations are a primary causal factor of systemic insulin resistance and metabolic disorder [12]. Therefore, MG53 may possess a cardioprotective function [13]. MG53 may also have myokine functions for tissue protection [14]. MG53 is predominantly localized in small vesicles underneath the plasma membrane, which are important for the quality control of KV2.1 in cardiomyocytes [15]. MG53 has promiscuity of function in the context of both normal and diseased hearts [16]. MG53 also induces insulin receptor substrate-1 (IRS-1) ubiquitination and degradation through the MG53-IRS-1 interaction in skeletal muscle [17]. MG53 may initiate the assembly of membrane repair machinery in an oxidation-dependent manner [18].

In this study, we aimed to explore the effect of RS on myocarditis in a model of LPS-induced inflammation injury in H9C2 cardiomyocytes in vitro. We hypothesized that MG53 might act as a myokine/cardiokine, contributing to RS sensitivity in myocarditis [19].

## Methods

### Cell lines and cell culture

Rat H9C2 cells were obtained from Xiamen Immocell Biotechnology Co., Ltd., China. H9C2 cells were cultured in PRIM1640 containing 5% fetal bovine serum (FBS), 100 U/mL penicillin and streptomycin, and 2 mM L-glutamine at 37 °C in a 5% CO_2_ incubator.

*LPS treatment.* 1 × 10^5^ Cells were treated with LPS (#L2630, Sigma. Co., Ltd) at a range of concentrations 0, 5, 10, 20 μg/ml for 24 h for subsequent cell functional assay. 10 μg/ml was selected for further measurement.

### Cell proliferation assay

A total of 4 × 10^3^ cells/well were seeded in 96-well plates. Cell proliferation was measured using an MTT kit (Cat: QF0025, Qiancheng Biotech, Shanghai) at each time point or drug concentration for 60 min at 37 °C. Each experiment was performed in sextuple.

### Detection of mitochondrial membrane potential

A JC-1 staining assay kit (C2006, Beyotime) was used to detect mitochondrial membrane potential. 1 × 10^6^ cells were resuspended in 200 µL of medium and then mixed with 200 µL of the JC-1 working solution. After incubation for 20 min at 37 °C in the dark, cells were washed twice with the JC-1 staining buffer, resuspended in 300 µL of the JC-1 staining buffer, and analyzed using a flow cytometer NovoCyte FACS (Cat#: 1300, ACEA, San Diego, CA, USA). Green (JC-1 monomer) and red (JC-1 aggregates) fluorescence were detected within the FITC-channel (Ex: 488 nm/Em: 519 nm) and PE-channel (Ex: 488 nm/Em: 578 nm), respectively. The mean fluorescence intensity (MFI) was measured, and then the MFI ratio of green/red was counted.

### Apoptosis assay

1 × 10^5^/100 μL cells were stained with 5 μL Annexin V-fluorescein isothiocyanate (FITC) and 5μL PI (Cat: A211-02, Vazyme, Nanjing, China) at 28 °C for 10 min according to the manufacturer’s introduction. Then the stained cells were subjected to the cytometer NovoCyte FACS and 1 × 10^4^ cells were analyzed within the FITC-channel (Ex: 488 nm/Em: 519 nm) and PE-channel (Ex: 488 nm/Em: 578 nm). Annexin V^+^/PI^−^ cells were indicated as early-apoptotic cells and annexin V^+^/PI^+^ cells were indicated as late-apoptotic cells. The results were represented as the percentage of apoptotic cells out of all analyzed cells.

### ELISA assay

Cell supernatants were collected and centrifuged with 500 g × 5 min. And the supernatant was collected for ELISA testing according to the instruction of kits, whose detailed information was shown in Table 1.

**Table 1:** Primers for qPCR

qPCR primers	Sequence (5′-3′)
18s forward primer 18s reverse primer	aggcgcgcaaattacccaatcc
	gccctccaattgttcctcgttaag
MG53 forward primer MG53 reverse primer	TGTTAAGCCTGTTCCTGACTG
	ATGGTGAGCAGGTCTGGT
IL-1β forward primer IL-1β reverse primer	CCTGAACTCAACTGTGAA
	TGGAAGCAATCCTTAATCT
IL-6 forward primer IL-6 reverse primer	GGAAATGAGAAAAGAGTTGTG
	AGAAGACCAGAGCAGATT
TNF-α forward primer TNF-α reverse primer	AACAAGGAGGAGAAGTTC
	TTGAGAAGATGATCTGAGT
MCP-1 forward primer MCP-1 reverse primer	AATGAGTCGGCTGGAGAA
	GCTTGGTGACAAATACTACAG

### Reverse transcription-quantitative PCR (RT-qPCR) assay

Total RNA extracted from cells using the RNA Isolater Total RNA Extraction Reagent (Vazyme, Nanjing, Jiangsu, China) was subjected to reverse transcription using Superscript III Reverse Transcriptase (Invitrogen, Thermo Fisher Scientific, Inc.) at 47 °C for 50 min. The qPCR assay was conducted using the ChamQ SYBR® qPCR Master Mix (Vazyme Biotech Co., Ltd.). The thermocycling conditions were as follows: 98 °C for 30 s, followed by 40 cycles of 98 °C for 5 s, 60 °C for 15 s. Each reaction was performed in triplicate. The 2^−ΔΔCt^ method was used to analyze the expression levels of genes, which were normalized to 18S ribosomal RNA. The qPCR primers are listed in Table 2.

**Table 2:** Kit and antibody list

Kit or antibody	Supplier	Cat#	Dilution
ClonExpress Ultra One Step Cloning Kit	Vazyme	C115-01	NA
Rat IL-1β/IL-1F2 Immunoassay	R&D	RLB00	NA
Rat IL-6 Immunoassay	R&D	R6000B	NA
Rat IL-10 Immunoassay	R&D	R1000	NA
Rat CCL2/JE/MCP-1 Immunoassay	R&D	DY3144-05	NA
Rat TNF-α Immunoassay	R&D	RTA00	NA
GAPDH	Proteintech	10494-1-AP	1:3000
MG53	Proteintech	22151-1-AP	1:5000
Bcl-2	Abcam	ab194583	1:1000
Bax	Abcam	ab232479	1:1000
Cleaved caspase 3	Cell Signaling Technology	9661	1:1000
Cleaved caspase 9	Cell Signaling Technology	9507	1:1000
Cyclin D1	Abcam	ab134175	1:1000
Cyclin A1	Abcam	ab53699	1:1000
Cyclin E1	Proteintech	11554-1-AP	1:1000
CDK2	Proteintech	10122-1-AP	1:2000
P65	Proteintech	66535-1-Ig	1:3000
P-P65	Abcam	ab76302	1:1000
IkBa	Proteintech	10268-1-AP	1:2000
p- IkBa	Cell Signaling Technology	9246	1:1000
HRP Goat Anti-Rabbit IgG(H + L) HRP Goat Anti-Mouse IgG(H + L)	Proteintech Proteintech	SA00001-2 SA00001-1	1:10,000
			1:10,000

### Western blotting

Cells were lysed in chilled lysis buffer (Sangon Biotech Co., Ltd.), and total protein was quantitated using the BCA Protein Assay kit (Abcam). Lysates (10 μg/sample) were resolved using 10% denaturing SDS-PAGE and the proteins were transferred onto a PVDF membrane (Millipore). Tris–HCl buffer containing 5% bovine serum albumin (BSA; Beijing Solarbio Science & Technology Co., Ltd.) was used to block the membranes at 37 °C for 1 h. The membrane was probed with the respective primary antibodies prepared in Tris–HCl buffer containing 5% BSA and incubated overnight at 4 °C. Subsequently, the membranes were washed, followed by incubation with the corresponding secondary antibodies prepared in Tris–HCl buffer at 28 °C for 1 h. Detailed information on the antibodies is shown in Table 1. The bands were visualized using chemiluminescence detection reagent (Thermo Fisher Scientific, Carlsbad, CA, USA). All the experiments were performed three times, and the bands were semi-quantified via densitometry using ImageJ 1.52v (NIH, Bethesda, MD, USA). GAPDH was used as loading control.

### siRNA transfection

The siRNA-1, siRNA-2, siRNA-3 was synthesized from the GenScript Biotech (Nanjing, China) based on the sequence: 5′-GACUGAGUUCCUCAUGAAAUATT-3′; 5′-CGCUGAGCAUCUACUGCGAGCTT-3′; 5′-CGCUGUGCCUGCAGCUGUUCGTT-3′. H9C2 were seeded into 6-well plates at a density of 2 × 10^6^ per well. The cells were transfected with siRNA (short interfering RNA) using Lipofectamine 2000 (Invitrogen; Thermo Fisher Scientific, Inc.) at 37 °C when they reached 80% confluence. The cells were lysed 48 h post-transfection, and MG53 expression was analyzed using western blotting and RT-qPCR. Each experiment was conducted in triplicate*.* The cells were also treated with 10 μg/mL LPS and 1.0 μM Rosuvastatin (#B1123, Apexbio. Co., Ltd) 12 h post-transfection in the rescue experiments.

### Statistical analysis

All statistical analyses were conducted using SPSS version 22.0 (IBM Corp.) and GraphPad Prism version 8.0.2 (GraphPad Software, Inc.). Data are presented as mean ± standard deviation. Differences between two groups were analyzed using the Student’s *t*-test. ANOVA followed by the Tukey’s post-hoc test was used for multiple comparisons among three or more experimental groups. The level of statistical significance was set at *P* < 0.05.

## Results

### Development of in vitro myocarditis model of LPS-treated H9C2 cells

**Fig. 1:**
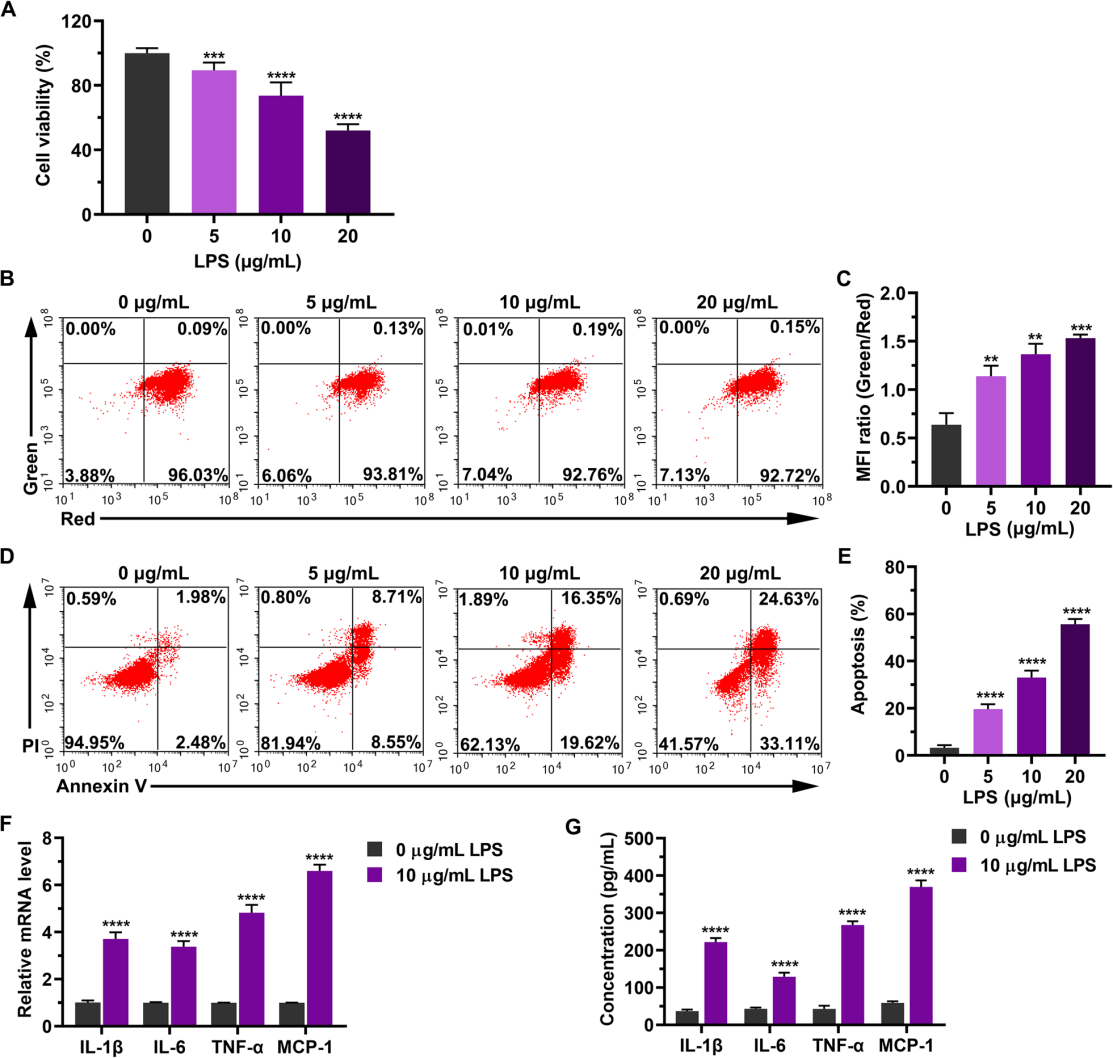
Construction of in vitro myocarditis model of LPS-treated H9C2 cells. H9C2 cells were treated with different concentrations of LPS for 24 h. **A** Cell viability was analyzed using the MTT assay and was calculated as a percentage, compared with the corresponding blank control. Data are represented as mean ± standard deviation (SD) of six biological replicates. **B** Mitochondrial membrane potential was determined using JC-1 assay. Representative images from three independent experiments. **C** Quantification of the mitochondrial membrane potential level indicated using mean fluorescence intensity (MFI). **D** Cell apoptosis was analyzed through Annexin-V/PI staining via FACS. Representative images from three independent experiments. **E** Quantification of apoptosis. **F** Quantitation of the mRNA expression of cytokines, namely IL-1β, IL-6, TNF-α, and MCP-1, as estimated using RT-qPCR. Data are represented as mean ± SD of three technical replicates. **G** Quantitation of level of cytokines, namely IL-1β, IL-6, TNF-α, and MCP-1, as determined using ELISA. Data are represented as mean ± SD of three biological replicates. Group differences were analyzed using one-way ANOVA with the Tukey’s post-hoc test, compared with 0 μg/mL LPS group. **P < 0.01; ***P < 0.005; ****P < 0.001

To mimic the conditions of myocarditis, H9C2 cells were treated with different concentrations of LPS for 24 h. We then examined the H9C2 cell status and inflammatory markers in cardiomyocytes in response to LPS stimulation in vitro. Cell viability was significantly reduced with increasing LPS concentration (Fig. 1A). The mitochondrial membrane potential was determined using JC-1 assay. An increase in MFI ratio (Green/Red) indicated a decrease in mitochondrial membrane potential. As shown in Fig. 1B, C, LPS treatment reduced the mitochondrial membrane potential in a concentration-dependent manner. As LPS induced cardiomyocyte apoptosis [20], the percentage of apoptotic cells was significantly increased in the LPS-treated group (Fig. 1D, E). Additionally, we also detected inflammatory markers because LPS could increase serum levels of interleukin-1β (IL-1β), interleukin-6 (IL-6), tumor necrosis factor-α (TNF-α), and monocyte chemoattractant protein-1 (MCP-1) [21]. We found that the mRNA levels of IL-1β, IL-6, TNF-α, and MCP-1, as assessed using RT-qPCR, were significantly increased by LPS stimulation (Fig. 1F, G). Furthermore, our results also indicated that LPS exposure increased cytokine levels of IL-1beta, IL-6, TNF-α, and MCP-1 as measured by ELISA (Fig. 1G). Taken together, our results demonstrate that LPS treatment reduces cell viability, increases apoptosis, and promotes pro-inflammatory cytokine expression in H9C2 cells.

### RS ameliorates LPS-induced inflammatory injury

**Fig. 2:**
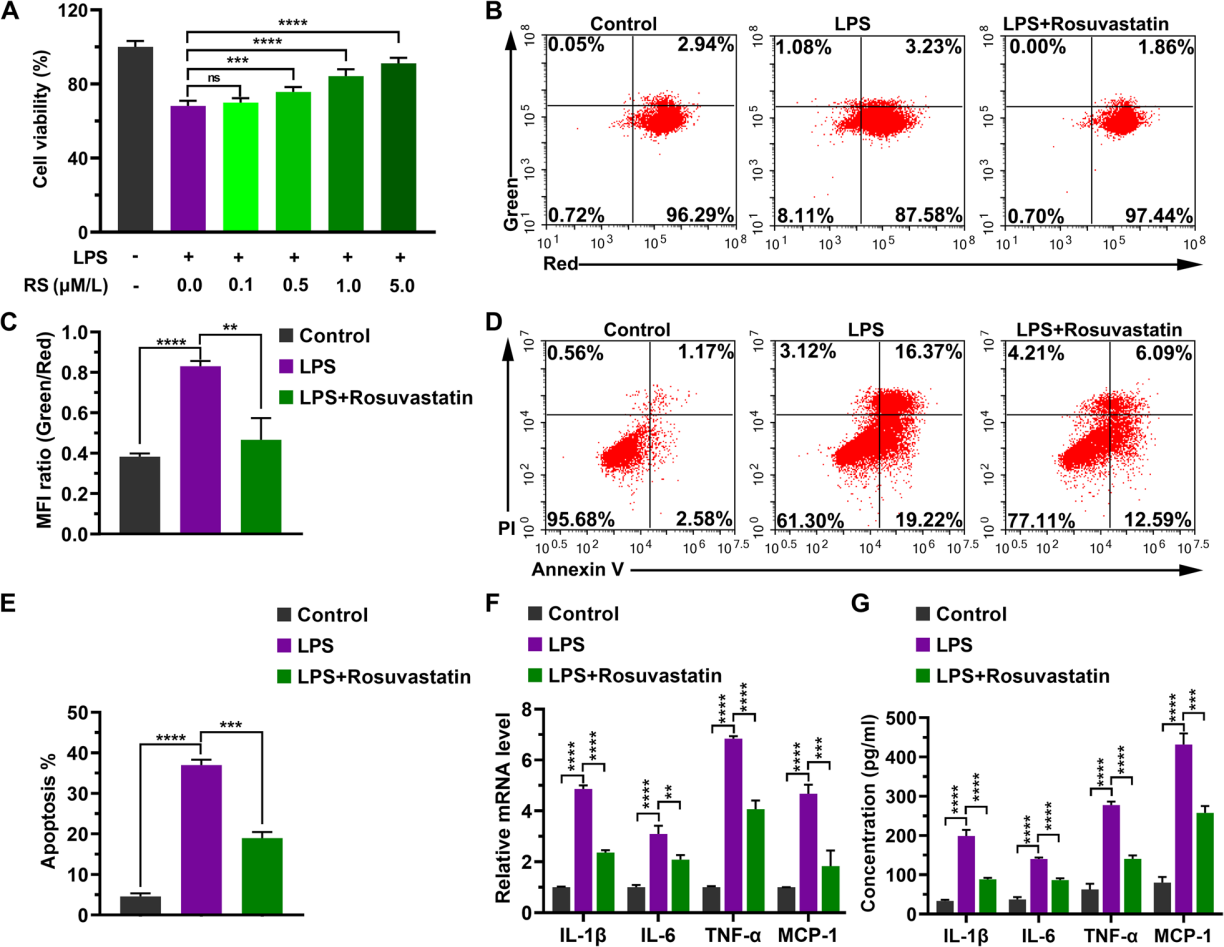
Effect of LPS on cell viability in H9C2 cells with or without RS. **A** Cell viability was analyzed and calculated as percentage. H9C2 cells were treated with 10 μg/ml LPS and different concentrations of RS for 24 h. Data are represented as mean ± SD of six biological replicates. RS: Rosuvastatin. **B** Mitochondrial membrane potential was determined using JC-1 assay. Representative images from three independent experiments. **C** Quantitation of mitochondrial membrane potential level indicated using MFI. **D** Cell apoptosis was analyzed using Annexin-V/PI staining via FACS. Representative images from three independent experiments. **E** Quantitation of apoptotic cells. **F** Quantitation of mRNA expression of cytokines, namely IL-1β, IL-6, TNF-a, and MCP-1, as estimated by RT-qPCR. Each experiment was performed in triple. **G** Quantitation of the level of cytokines, namely IL-1β, IL-6, TNF-a, and MCP-1, as determined using ELISA Data are represented as mean ± SD of three biological replicates. H9C2 cells were treated with 10 μg/mL LPS and 1.0 μM RS for 24 h in (**B**–**G**). Group differences were analyzed using one-way ANOVA with the Tukey’s post-hoc test. ns, not significant; **P < 0.01; ***P < 0.005; ****P < 0.001

To investigate the role of RS in LPS-induced cardiomyocyte injury, we analyzed LPS treated-H9C2 cells after RS treatment. First, we investigated the effect of RS on restoring the cell viability. As shown in Fig. 2A, LPS treatment significantly reduced the cell viability compared to control group (68.1% ± 2.8% vs 100.0% ± 3.2%, *P* < 0.0001). The LPS-induced impairment in cell viability was evidently reversed by treatment with 0.5 μM (75.7% ± 2.6%, *P* = 0.0015), 1.0 μM (84.3% ± 3.6%, *P* < 0.0001), and 5.0 μM (91.1% ± 3.1%, *P* < 0.0001) RS except for 0.1 μM RS (69.9% ± 2.3%, *P* = 0.8864) (Fig. 2A). Second, we found that RS significantly reversed the LPS-induced decrease in mitochondrial membrane potential. The MFI ratios of group “LPS” and group “LPS + Rosuvastatin” were 0.83 ± 0.02 and 0.46 ± 0.11, respectively (*P* = 0.0011, Fig. 2B, C). We also found that RS ameliorated the LPS-induced increase in percentage of apoptotic cells (19.0% ± 1.5% vs 36.96% ± 1.3%, *P* < 0.0001, Fig. 2D, E). Additionally, we found that RS reduced the mRNA levels of cytokines IL-1β, IL-6, TNF-α, and MCP-1 (Additional file 1: Supplementary Table 1, Fig. 2F). Furthermore, as shown in Fig. 2G, we found that RS reversed the secretory level of IL-1β, IL-6, TNF-α, and MCP-1, which was increased by LPS treatment (Additional file 1: Supplementary Table 2, Fig. 2G). In summary, RS pretreatment significantly reduces LPS-induced cell injury, increases cell viability, reduces cell apoptosis, and inhibits the expression of pro-inflammatory factors.

### RS enhances the expression of MG53 while MG53 deficiency exacerbates LPS-induced inflammatory injury

**Fig. 3:**
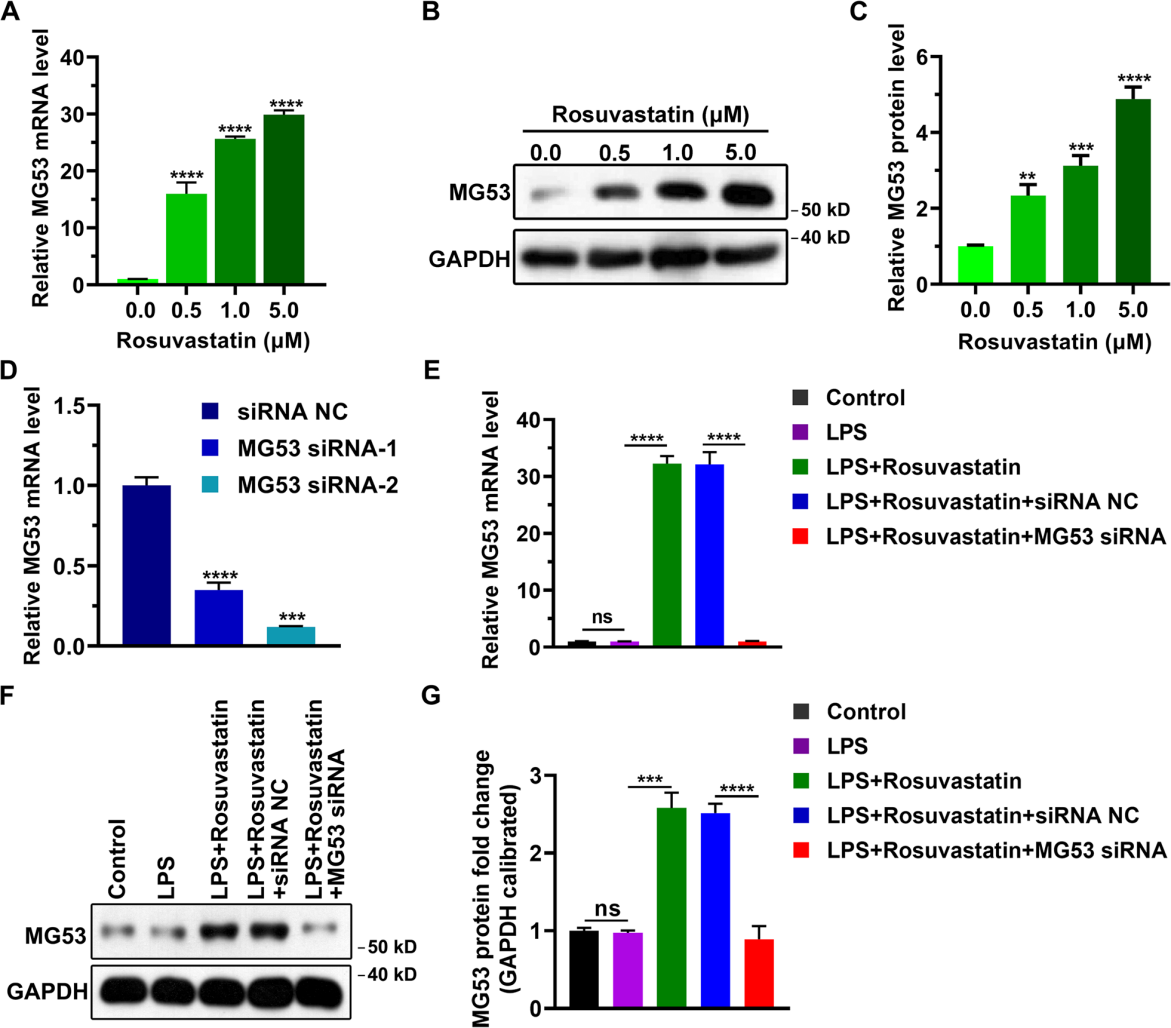
Effect of RS on MG53 expression under conditions of LPS treatment. **A** RS increased MG53 mRNA level in a concentration-dependent manner. RT-qPCR was performed when H9C2 cells were treated with different concentrations of RS for 24 h. Data are represented as mean ± SD of three technical replicates. **B** MG53 protein levels were detected using western blotting after treatment with RS for 24 h. Representative image of three independent experiments. **C** Quantification of (**B**). **D** The knockdown efficiencies of siRNAs were determined using RT-qPCR. Data are represented as mean ± SD of three technical replicates. (E) MG53 mRNA expression as analyzed in the following five groups: 1) Control; 2) Treatment with 10 μg/mL LPS for 24 h; 3) Combined treatment with 10 μg/mL LPS and 1.0 μM RS for 24 h; 4) Combined treatment for 24 h after a 12-h transfection with siRNA negative control; and 5) Combined treatment for 24 h after a 12-h transfection with MG53 siRNA. Data are presented as mean ± SD of three technical replicates. **F** MG53 protein expression as analyzed using western blotting. Representative image of three independent experiments. **G** Quantification of MG53 protein. Group differences were analyzed using one-way ANOVA with the Tukey’s post-hoc test. ns, not significant. **P < 0.01, ***P < 0.005, ****P < 0.001

To investigate the mechanism of RS-mediated protective effect in LPS-induced injury, we first examined the expression of MG53 after treatment with RS, since MG53 protects cells against LPS-induced injury [19]. As shown in Fig. 3A, MG53 mRNA expression in H9C2 cells was increased with increasing concentrations of RS. Additionally, the protein level of MG53 was also increased by RS treatment in a concentration-dependent manner (Fig. 3B, C). To further examine the functional roles of MG53 in LPS-induced in vitro, MG53 expression was inhibited by transfection with siRNAs targeting MG53. As shown in Fig. 3D, MG53 mRNA level was significantly reduced by MG53 siRNAs in H9C2 cells, and MG53 siRNA-2 was selected for subsequent experiment. MG53 mRNA and protein levels were increased after treatment with RS compared to the single LPS-injured group (Fig. 3E, G), while MG53 silence reversed this increase (Fig. 3E, G).

### The protective effect of RS on H9C2 cells with LPS-induced injury is blocked by MG53 deficiency

**Fig. 4:**
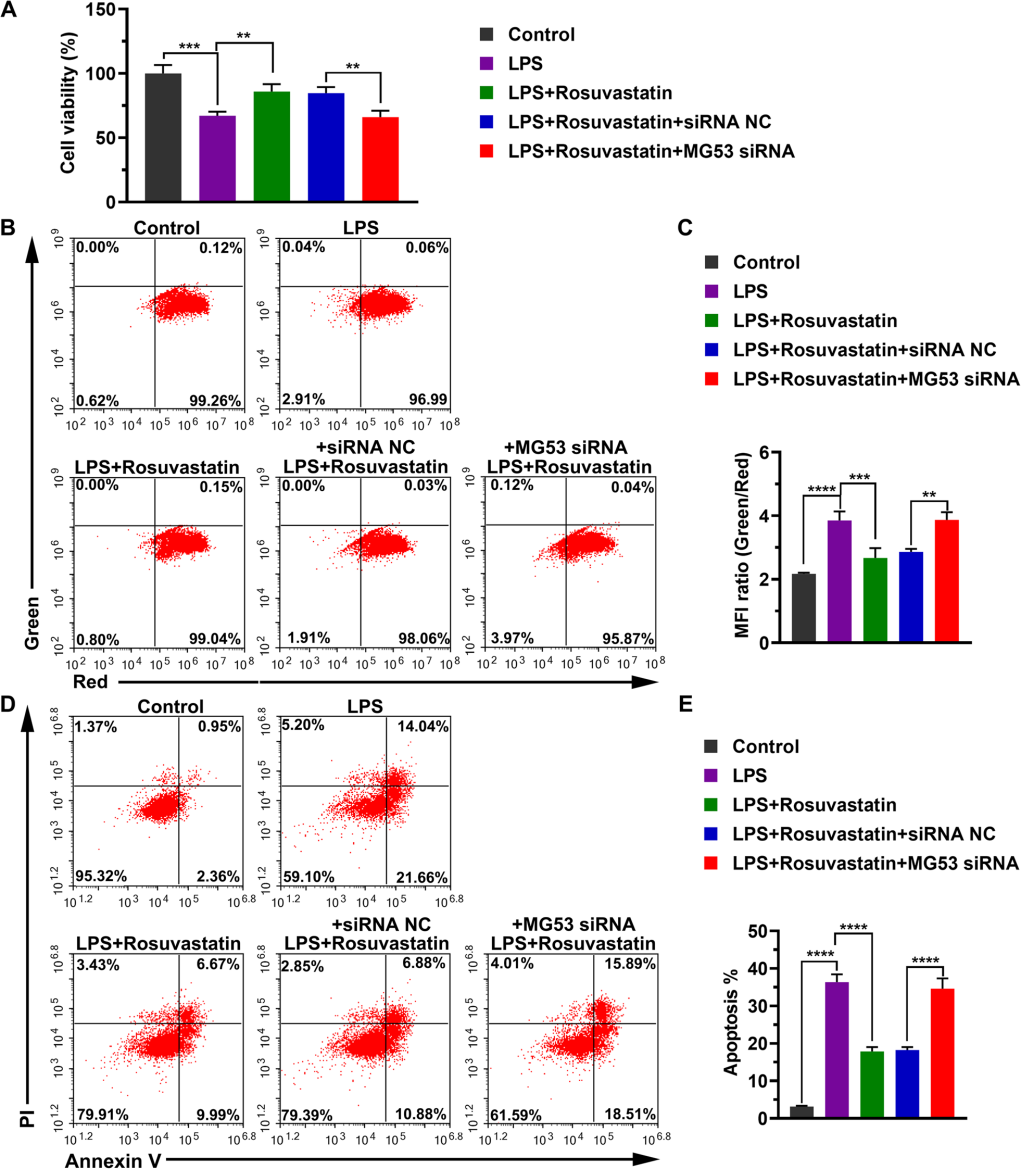
MG53 deficiency eliminates the protective effect of Rosuvastatin on H9C2 cells. **A** Cell viability was measured by an MTT assay and was calculated as percentage compared with the corresponding group. Data are represented as mean ± SD of six biological replicates. **B** Mitochondrial membrane potential was analyzed using JC-1 assay. Representative images from three independent experiments. **C** Quantitation of the mitochondrial membrane potential level indicated using MFI. **D** Cell apoptosis was analyzed using Annexin-V/PI. Representative images from three independent experiments. **E** Quantitation of apoptotic cells. Group differences were analyzed using one-way ANOVA with the Tukey’s post-hoc test. *P < 0.05, **P < 0.01, ***P < 0.005, ****P < 0.001

**Fig. 5:**
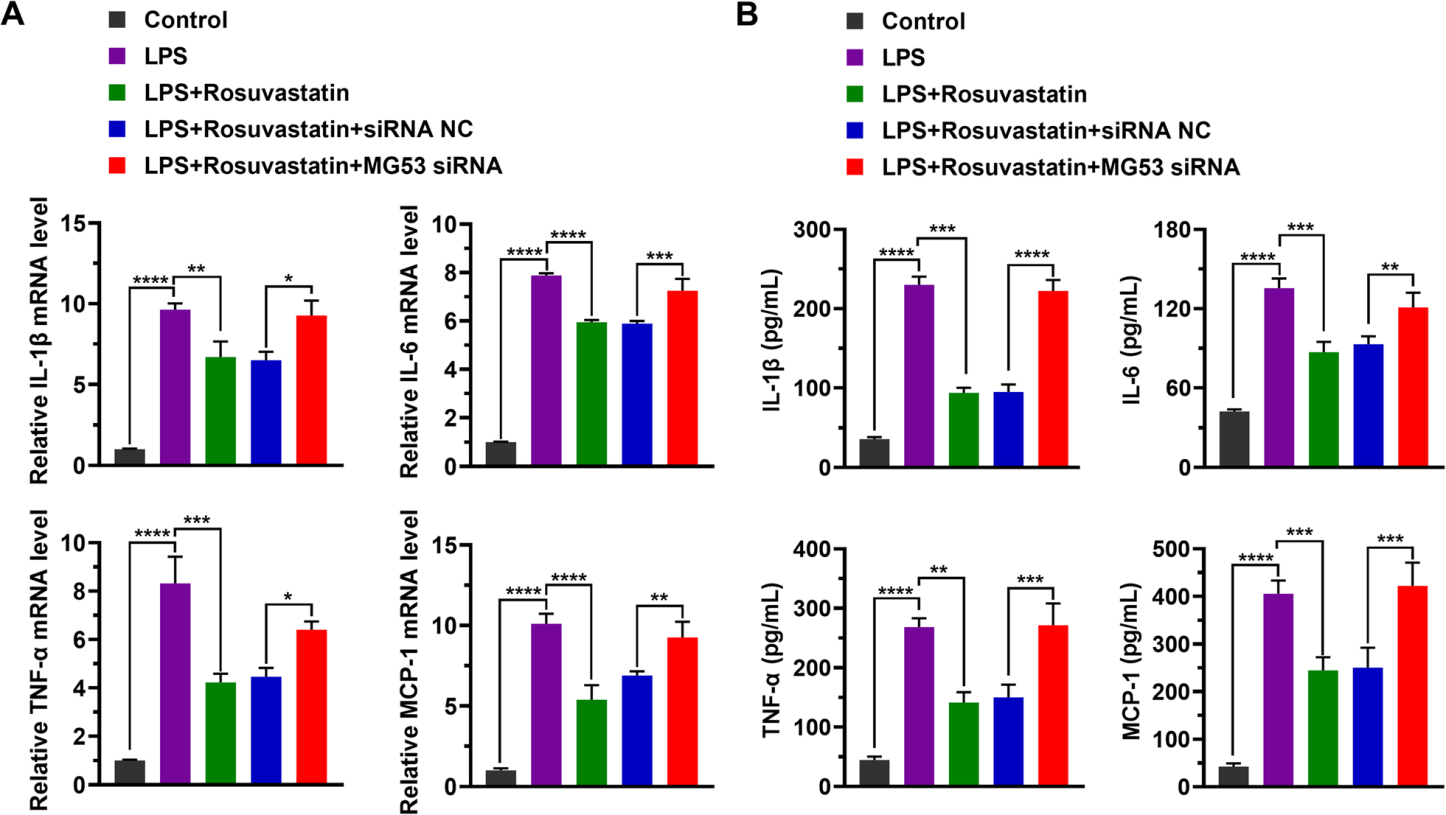
The Rosuvastatin-induced decrease in cytokine level was abolished by MG53 knockdown. **A** Quantitation of the mRNA expression of cytokines, namely IL-1β, IL-6, TNF-a, and MCP-1, as analyzed using RT-qPCR. Data are represented as mean ± SD of three technical replicates. **B** Quantitation of cytokine levels using ELISA. Data are represented as mean ± SD of three biological replicates. Group differences were analyzed using one-way ANOVA with the Tukey’s post-hoc test. *P < 0.05, **P < 0.01, ***P < 0.005, ****P < 0.001

Next, we analyzed the effect of MG53 deficiency on the protective effect of RS in LPS-injured cells. We observed that MG53 silence significantly eliminated the enhanced effect of RS on cell viability (84.8% ± 4.8% vs 66.0% ± 5.0%, *P* = 0.0011, Fig. 4A). MG53 silence also re-reduced the mitochondrial membrane potential which was restored by RS after LPS-induced decrease. The MFI ratios of group “LPS + Rosuvastatin + siRNA NC” and group “LPS + Rosuvastatin + MG53 siRNA” were 2.86 ± 0.09 and 3.87 ± 0.23, respectively (*P* = 0.0016, Fig. 4B, C). As shown in Fig. 4D, E, RS significantly attenuated cell apoptosis induced by LPS (17.8% ± 1.2% vs 36.4% ± 2.1%, *P* < 0.0001). However, the effect of RS on reducing cell apoptosis was abolished when MG53 expression was down-regulated (18.2% ± 0.8% vs 34.6% ± 2.7%, Fig. 4D, E). Additionally, we observed that the effect of RS on the mRNA levels of cytokines IL-1β, IL-6, TNF-α, and MCP-1 were significantly attenuated by MG53 down-regulation (Additional file 1: Supplemental table 3, Fig. 5A). Moreover, similar changes in cytokine levels were observed (Additional file 1: Supplemental table 4, Fig. 5B).

### RS modulates multiple signaling pathways in an MG53-dependent manner in H9C2 cardiomyocytes with LPS-induced injury

**Fig. 6:**
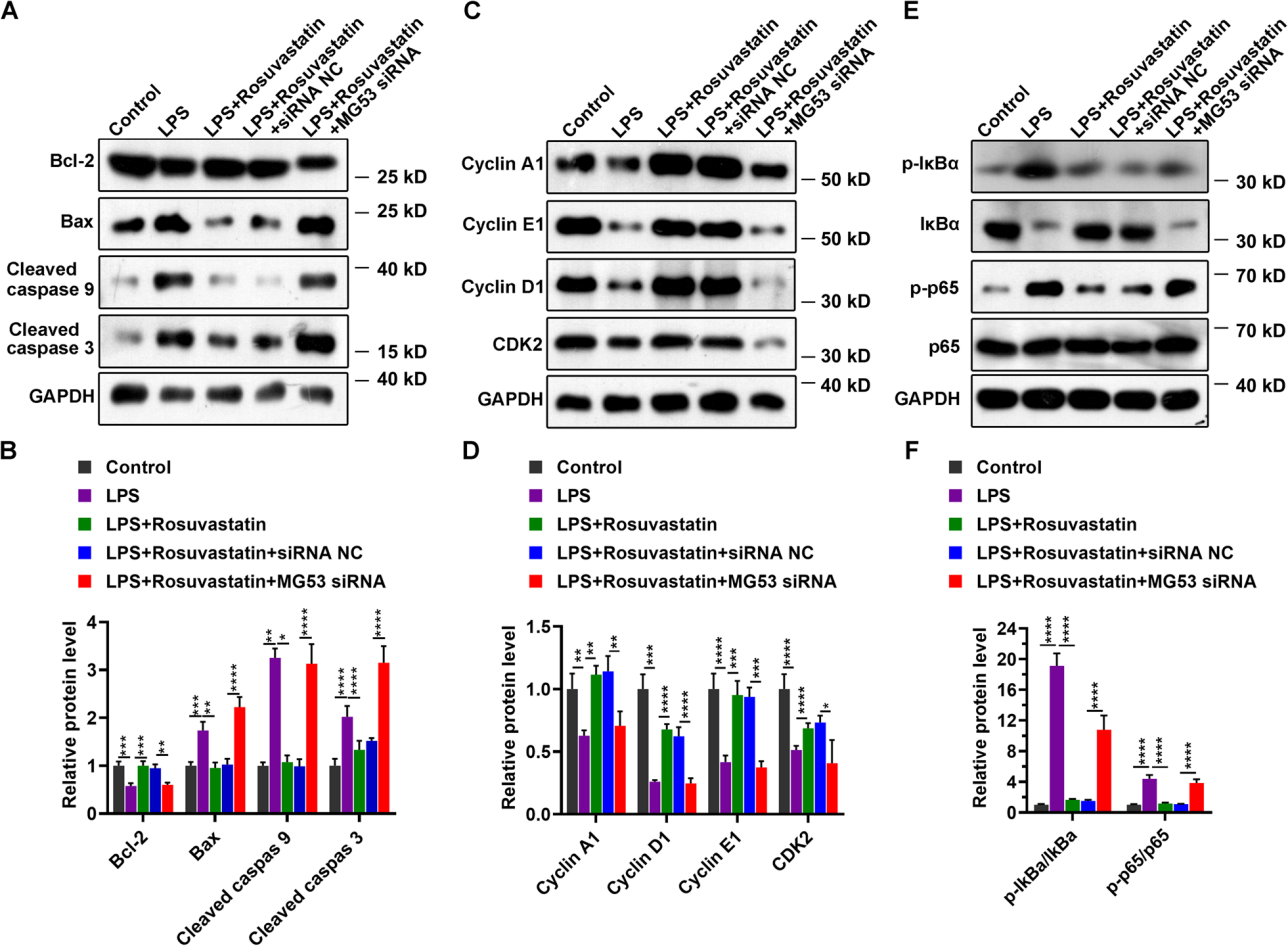
Effect of RS on signaling pathways under conditions of LPS treatment. **A**, **C**, **E** Protein expression as analyzed using western blotting. Representative image of three independent experiments. **B**, **D**, **F** Quantitation of proteins. Group differences were analyzed using one-way ANOVA with the Tukey’s post-hoc test. *P < 0.05, **P < 0.01, ***P < 0.005, ****P < 0.001

To investigate the potential molecular mechanisms involved in the protection of RS form LPS-induced injury in H9C2 cardiomyocytes, we explored the protein expression levels of the following proteins: cyclin A1, cyclin E1, cyclin D1, and CDK2, which modulate cell cycle progress; cell-apoptosis related proteins such as Bcl-2, Bax, cleaved caspase 9, and cleaved caspase3; NF-κB signaling pathway proteins, namely p-IκBa, Iκba, p-p65, and p65. As shown in Fig. 6, LPS treatment remarkably increased the protein levels of Bax, cleaved caspase 9, cleaved caspase 3, p-IkBa, and p-p65, and reduced the expression of Bcl-2, cyclin A1, cyclin E1, cyclin D1, and CDK2. Moreover, we found that RS partially reversed the effect of LPS on these proteins. As we expected, MG53 knockdown increased the protein levels of cleaved caspase 9, cleaved caspase 3, cyclin D1, CDK2, and p-p65 (Additional file 1: Supplementary Fig. 1). Finally, we detected the levels of above proteins in H9C2 cells, which were treated with LPS after RS treatment combined with or without MG53 knockdown. As shown in Fig. 6, downregulation of MG53 attenuated the effect of RS, compared with the group treated with RS alone.

## Discussion

Primary cardiomyocytes from rat neonatal hearts are widely used to investigate the cellular and molecular changes, which has an evident disadvantage that it needs high numbers of animals [22]. Thus, several cardiomyocyte cell lines, for example, H9C2, were established for study [23]. The H9C2 cell line was originally derived from embryonic rat ventricular tissue. Although H9C2 cells are no longer able to beat, they still show many similarities to primary cardiomyocytes, including membrane morphology, g-signalling protein expression and electrophysiological properties [24]. Importantly, they can display hypertrophy-associated traits when stimulated with hypertrophic agents in vitro [25–28]. Based on methods reported in a previous study [29], we successfully constructed an LPS-induced rat cardiomyocyte injury model using H9C2 cells in which LPS reduced cell viability, increased apoptotic cells, and promoted pro-inflammatory levels. However, use of H9C2 cells as an in vitro model of myocarditis has been open to question because they are a proliferating cell line, in contrast to the non-proliferating nature of primary cardiomyocytes. Gene expression profiling has previously demonstrated increased cell cycle and pro-survival protein expression such as cyclin A and Bcl-2 in undifferentiated myoblasts [30]. The extent to which H9C2 cells can accurately mimic responses of terminally differentiated cardiac myocytes is a limitation of our study. Moreover, validation of our findings should be performed in vivo.

The process of myocarditis might be caused by the activation of endoplasmic reticulum stress and autophagy [31]. Alternatively, it might be caused by the activation of NLRP3 inflammasome-mediated pyroptosis and related cytokine release [32]. Furthermore, the MALAT1-SAA3 signaling pathway was shown to enhance TNF-α expression in LPS-treated cardiomyocytes and could be involved in the phenotypes observed [33]. Our study indicated that LPS treatment may induce myocarditis via NF-κB signaling pathway. Wang K, et al. reported that rosuvastatin inhibits apoptosis of human coronary artery endothelial cells via JAK2/STAT3 signaling pathway [34]. Our study revealed that RS plays a protective role in H9C2 cells with LPS-induced injury though reversing NF-κB signaling pathway. Moreover, previous studies reported that RS enhances the expression of Bcl-2 and Cyclin D1, but reduces the expression of apoptotic cytokines and caspase-3 [11, 35, 36], which are consistent with our results. There are other drugs that may have similar effects with rosuvastatin. For example, GEN reduces the LPS-induced damage of cardiomyocytes [37]. Alprostadil increases the viability of LPS-stimulated H9C2 cells and attenuates IL-1β, IL-6, IL-17, and TNF-α secretion by modulating Wnt5a, JNK, and NF-κB expression [29]. Resveratrol exerts a therapeutic effect on LPS-induced inflammation via the TLR4 signaling pathway [38].

The possible mechanism involved in the protective effect of RS was further elucidated by knockdown of MG53. We observed that MG53 acted as a master regulator in this process. Serum MG53 is a biomarker of myocardial membrane injury [39]. Other studies also suggest that MG53 significantly mitigates neuroinflammation, as evidenced by reduced production of IL-1β and IL-6 in the hippocampus of LPS-treated mice [40]. Additionally, cholesterol-dependent MG53-mediated membrane repair is important for membrane function [21]. Furthermore, we observed that cell cycle regulators, apoptosis signaling pathway, and NF-κB pathway were modulated by the RS-MG53 axis in LPS-treated cells, which suggested that RS-MG53 targeted multiple signaling pathways to influence the host status.

In summary, our findings suggest important roles of RS for the protection of LPS-induced cardiomyocyte injury, which may function via an MG53-dependent mechanism though cell cycle and apoptosis progresses and NF-κB signaling pathway. These findings might reveal a novel potential drug target for myocarditis.

## Supplementary Information

**Additional file 1:** One-way ANOVA with the Tukey’s post-hoc test of cytokine levels, western blotting analysis of MG53 knockdown on the expression levels of signaling components supplementar, and raw data of western blotting.

## Data Availability

The datasets used and/or analyzed during the current study will be made available from the corresponding author on reasonable request.
