# Supplementary material for: Rosuvastatin exerts cardioprotective effect in lipopolysaccharide-mediated injury of cardiomyocytes in an MG53-dependent manner
Source: BMC Cardiovasc Disord. 2022 Feb 23;22:69. doi: 10.1186/s12872-022-02458-3 (PMC8865731; doi:10.1186/s12872-022-02458-3)

**Supplementary table 1.** Rosuvastatin reduced the mRNA level of cytokine

|  | Control | LPS | LPS+ Rosuvastatin |
| --- | --- | --- | --- |
| IL-1β | 1.00 ± 0.02 | 4.86 ± 0.14 ^****^ | 2.36 ± 0.09 ^ΔΔΔΔ^ |
| IL-6 | 1.00 ± 0.08 | 3.09 ± 0.32 ^****^ | 2.08 ± 0.18 ^ΔΔ^ |
| TNF-α | 1.00 ± 0.04 | 6.84 ± 0.09 ^****^ | 4.06 ± 0.34 ^ΔΔΔΔ^ |
| MCP-1 | 1.00 ± 0.01 | 4.67 ± 0.35 ^****^ | 1.83 ± 0.61 ^ΔΔΔ^ |

One-way ANOVA with the Tukey’s post-hoc test, n = 3. ****: vs Control, P < 0.0001. ^ΔΔ^: vs LPS, P < 0.01, ^ΔΔΔ^: P < 0.001, ^ΔΔΔΔ^: vs LPS, P < 0.0001.

**Supplementary table 2.** Rosuvastatin reduced level of secreted cytokine

| Cytokine (pg/mL) | Control | LPS | LPS+ Rosuvastatin |
| --- | --- | --- | --- |
| IL-1β | 33.4 ± 2.9 | 198.9 ± 15.6 ^****^ | 88.4 ± 4.0 ^ΔΔΔΔ^ |
| IL-6 | 37.1 ± 5.9 | 140.1 ± 3.9 ^****^ | 86.4 ± 4.7 ^ΔΔΔΔ^ |
| TNF-α | 62.5 ± 14.7 | 277.6 ± 9.1 ^****^ | 140.5 ± 8.7 ^ΔΔΔΔ^ |
| MCP-1 | 80.2 ± 14.4 | 431.8 ± 28.4 ^****^ | 257.9 ± 17.3 ^ΔΔΔ^ |

One-way ANOVA with the Tukey’s post-hoc test, n = 3. ****: vs Control, P < 0.0001. ^ΔΔΔ^: P < 0.001, ^ΔΔΔΔ^: vs LPS, P < 0.0001.

**Supplementary table 3.** The Rosuvastatin-induced decrease in cytokine mRNA level was abolished by MG53 knockdown

| Cytokine | Control | LPS | LPS+Rosuvastatin | LPS+Rosuvastatin  +siRNA NC | LPS+Rosuvastatin  +MG53 siRNA |
| --- | --- | --- | --- | --- | --- |
| IL-1β | 1.00 ± 0.04 | 9.64 ± 0.39 ^****^ | 6.69 ± 0.97 ^ΔΔ^ | 6.50 ± 0.51 | 9.27 ± 0.93 ^#^ |
| IL-6 | 1.00 ± 0.02 | 7.88 ± 0.10 ^****^ | 5.94 ± 0.09 ^ΔΔΔΔ^ | 5.89 ± 0.11 | 7.25 ± 0.49 ^###^ |
| TNF-α | 1.00 ± 0.04 | 8.32 ± 1.09 ^****^ | 4.26 ± 0.36 ^ΔΔΔ^ | 4.46 ± 0.37 | 6.41 ± 0.34 ^#^ |
| MCP-1 | 1.00 ± 0.13 | 10.10 ± 0.63 ^****^ | 5.40 ± 0.89 ^ΔΔΔΔ^ | 6.90 ± 0.26 | 9.26 ± 0.96 ^##^ |

One-way ANOVA with the Tukey’s post-hoc test, n = 3. ****: vs Control, P < 0.0001. ^ΔΔ^: vs LPS, P < 0.01. ^ΔΔΔ^: vs LPS, P < 0.001. ^ΔΔΔΔ^: vs LPS, P < 0.0001. ^#^: vs LPS+Rosuvastatin+siRNA NC, P < 0.05. ^##^: vs LPS+Rosuvastatin+siRNA NC, P < 0.01. ^###^: vs LPS+Rosuvastatin+siRNA NC, P < 0.001.

**Supplementary table 4.** The Rosuvastatin-induced decrease in secretary cytokine was abolished by MG53 knockdown

| Cytokine  (mg/mL) | Control | LPS | LPS+Rosuvastatin | LPS+Rosuvastatin  +siRNA NC | LPS+Rosuvastatin  +MG53 siRNA |
| --- | --- | --- | --- | --- | --- |
| IL-1β | 35.1 ± 2.0 | 230.5 ± 12.7 ^****^ | 96.83 ±2.7 ^ΔΔΔ^ | 98.8 ± 6.6 | 220.2 ± 16.2 ^####^ |
| IL-6 | 42.2 ± 1.5 | 135.5 ± 7.3 ^****^ | 87.1 ± 7.8 ^ΔΔΔ^ | 93.0 ± 6.1 | 120.9 ± 11.2 ^##^ |
| TNF-α | 44.8 ± 5.8 | 268.6 ± 14.6 ^****^ | 141.4 ± 17.5 ^ΔΔ^ | 150.1 ± 21.5 | 271.5 ± 36.7 ^###^ |
| MCP-1 | 1.00 ± 0.13 | 10.10 ± 0.63 ^****^ | 5.40 ± 0.89 ^ΔΔΔ^ | 6.90 ± 0.26 | 9.26 ± 0.96 ^###^ |

One-way ANOVA with the Tukey’s post-hoc test, n = 3. ****: vs Control, P < 0.0001. ^ΔΔ^: vs LPS, P < 0.01. ^ΔΔΔ^: vs LPS, P < 0.001. ^##^: vs LPS+Rosuvastatin+siRNA NC, P < 0.01. ^###^: vs LPS+Rosuvastatin+siRNA NC, P < 0.001. ^####^: vs LPS+Rosuvastatin+siRNA NC, P < 0.0001.


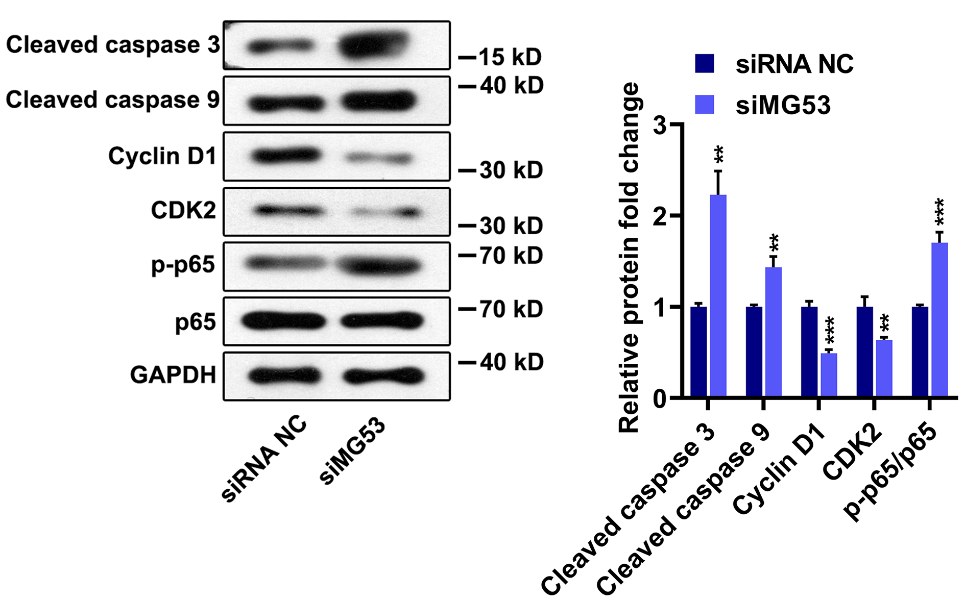


**Supplementary figure 1.** The effect of MG53 knockdown on the expression levels of signaling components.

gel figure 6A Bax


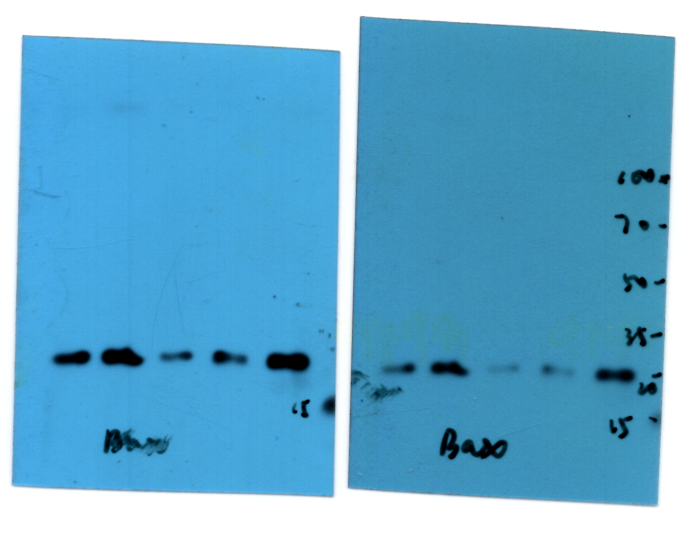


gel figure 6A Bcl-2


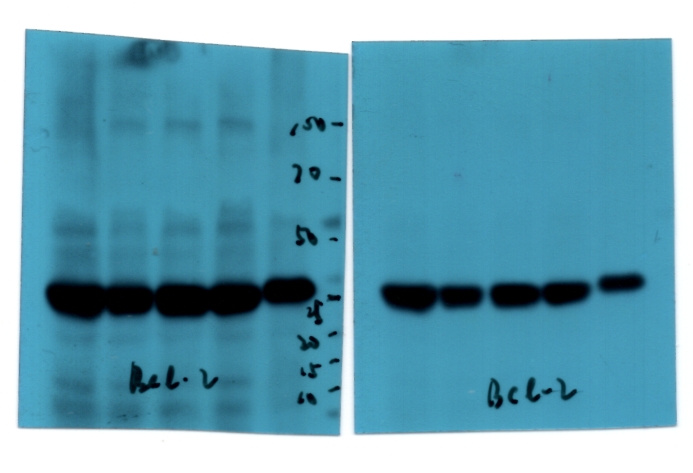


gel figure 6A cl-caspas3


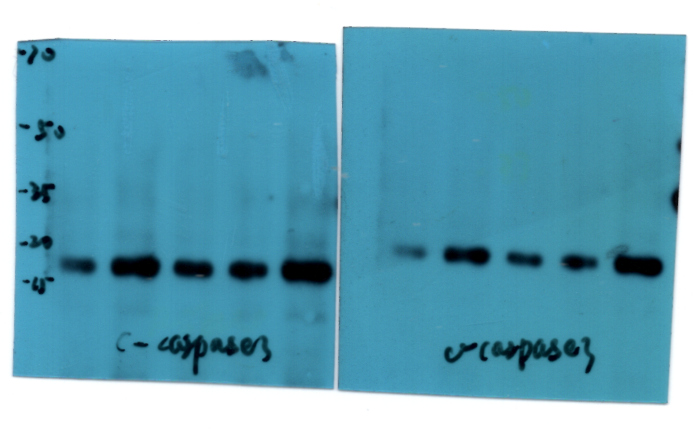


gel figure 6A cl-caspas9


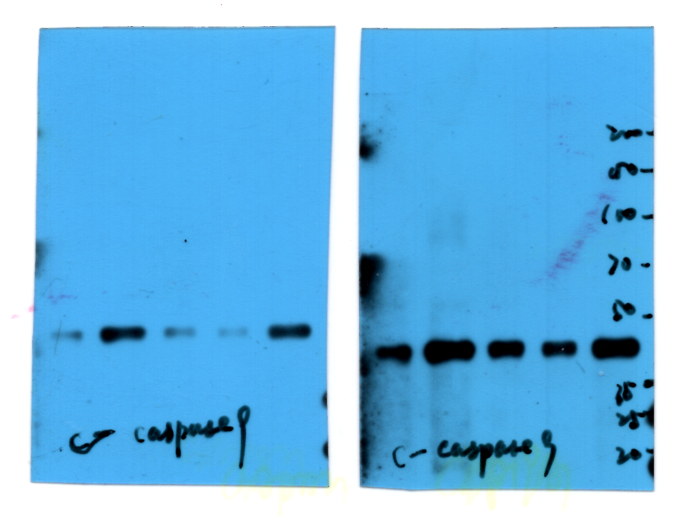


gel figure 6A GAPDH


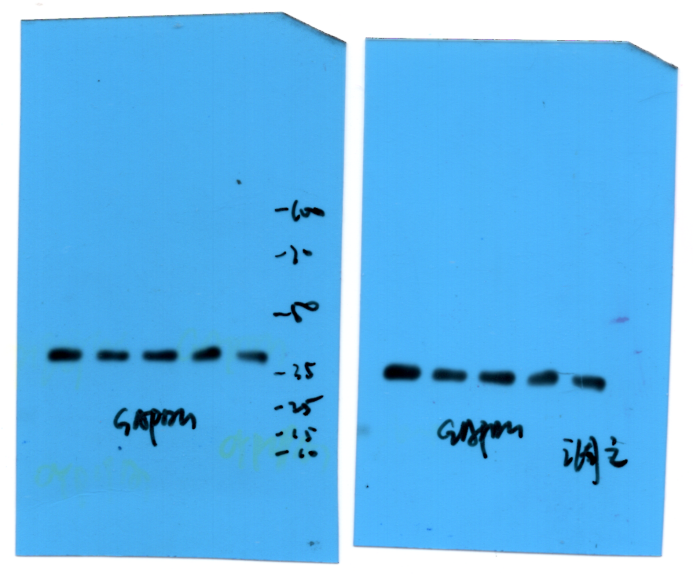


gel figure 6C CDK2


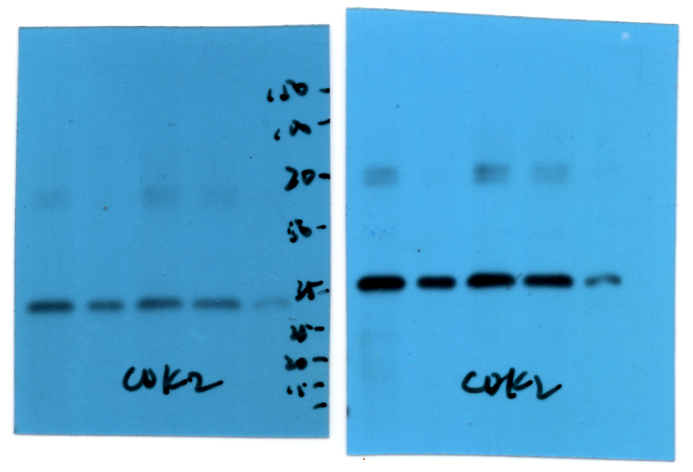


gel figure 6C cyclin A1


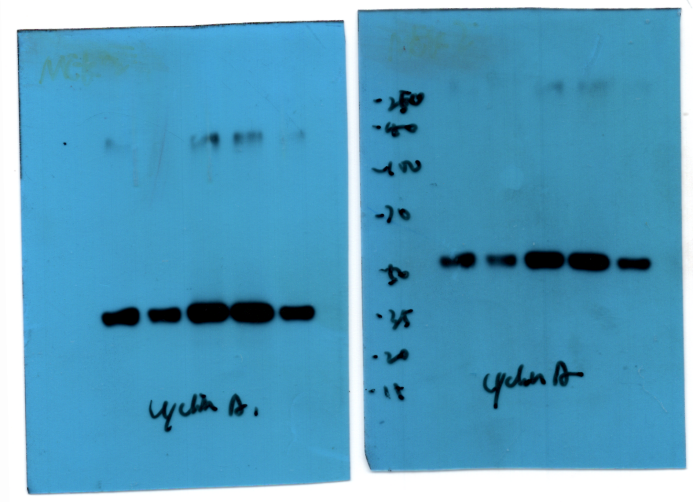


gel figure 6C cyclin D1


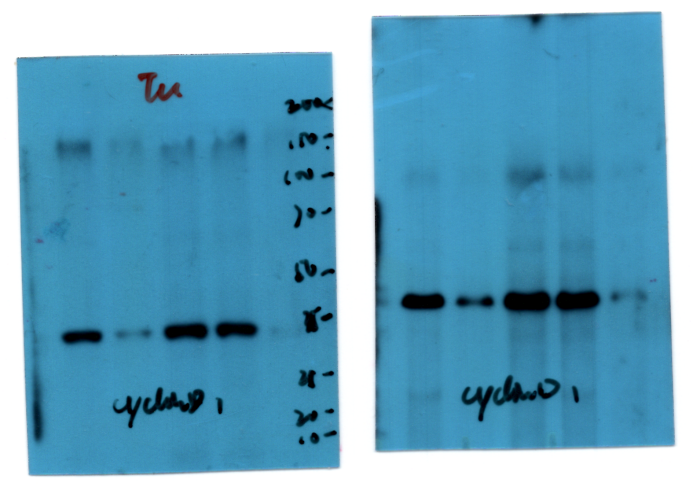


gel figure 6C cyclin E1


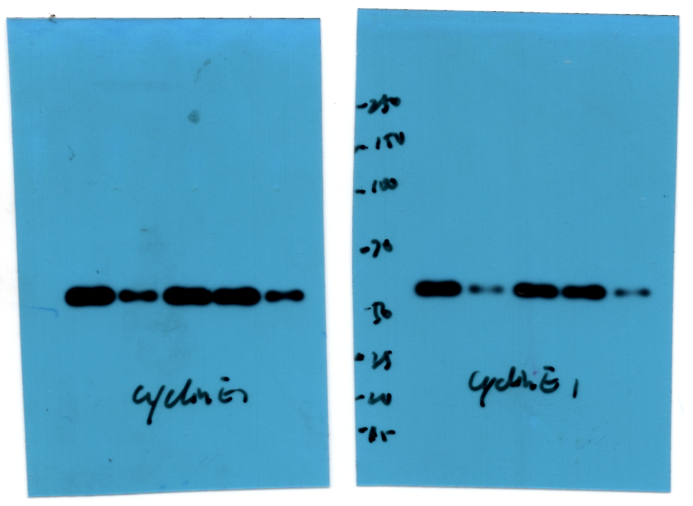


gel figure 6C GAPDH


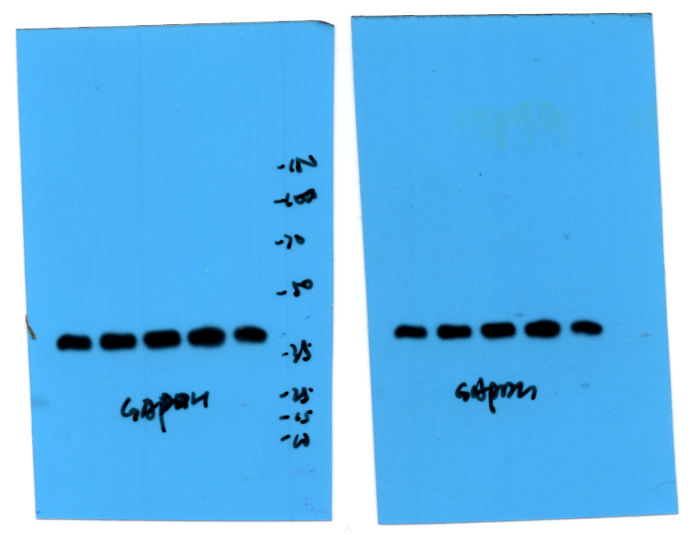


gel figure 6E GAPDH


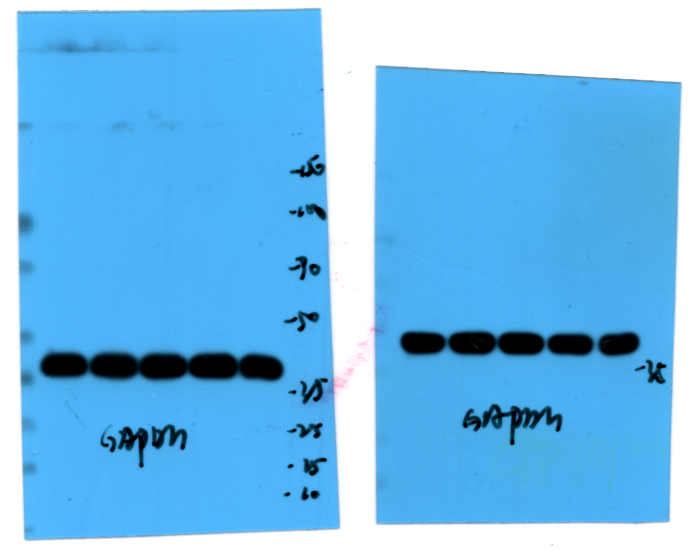


gel figure 6E IκBα


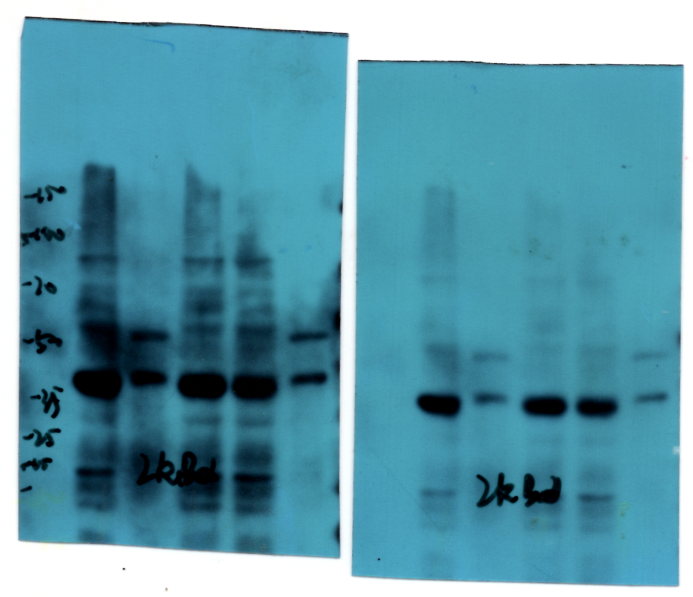


gel figure 6E p65


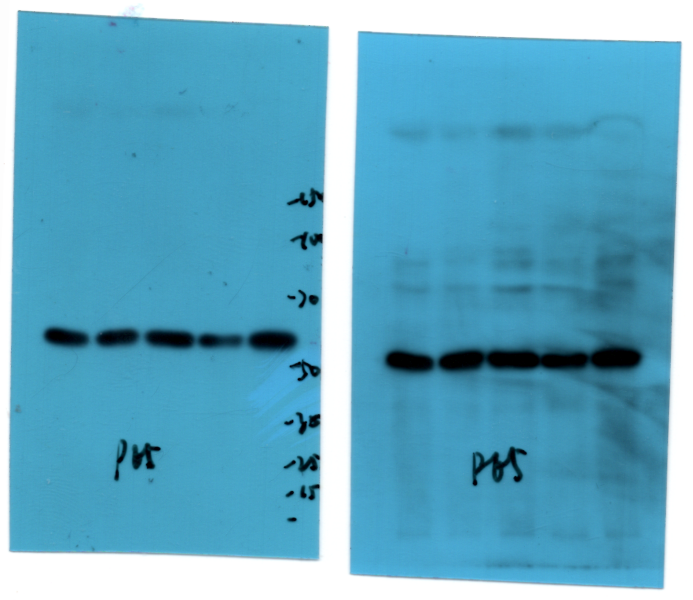


gel figure 6E p-IκBα


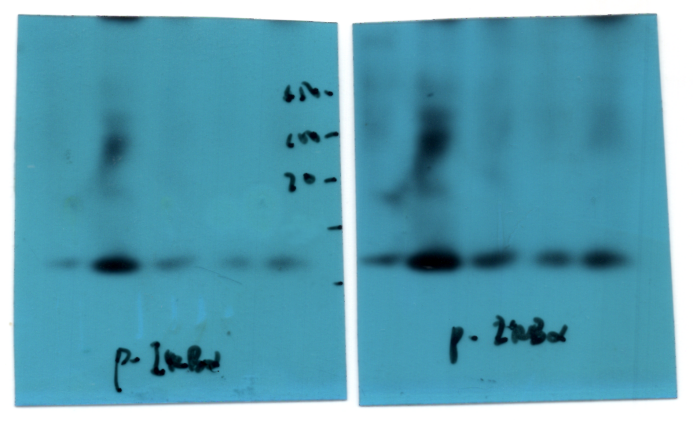


gel figure 6E p-p65


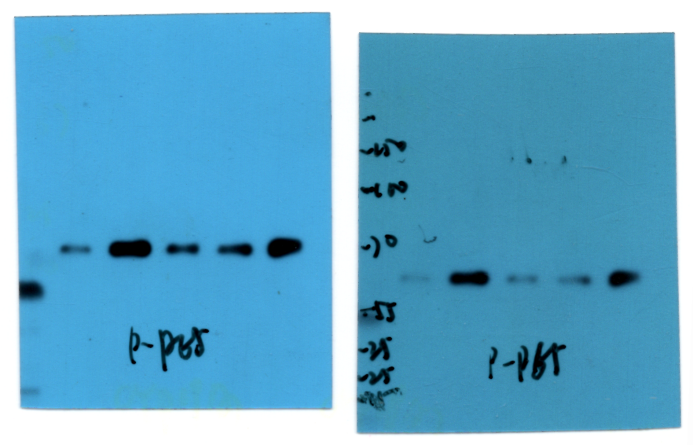


gel figure 3B GAPDH


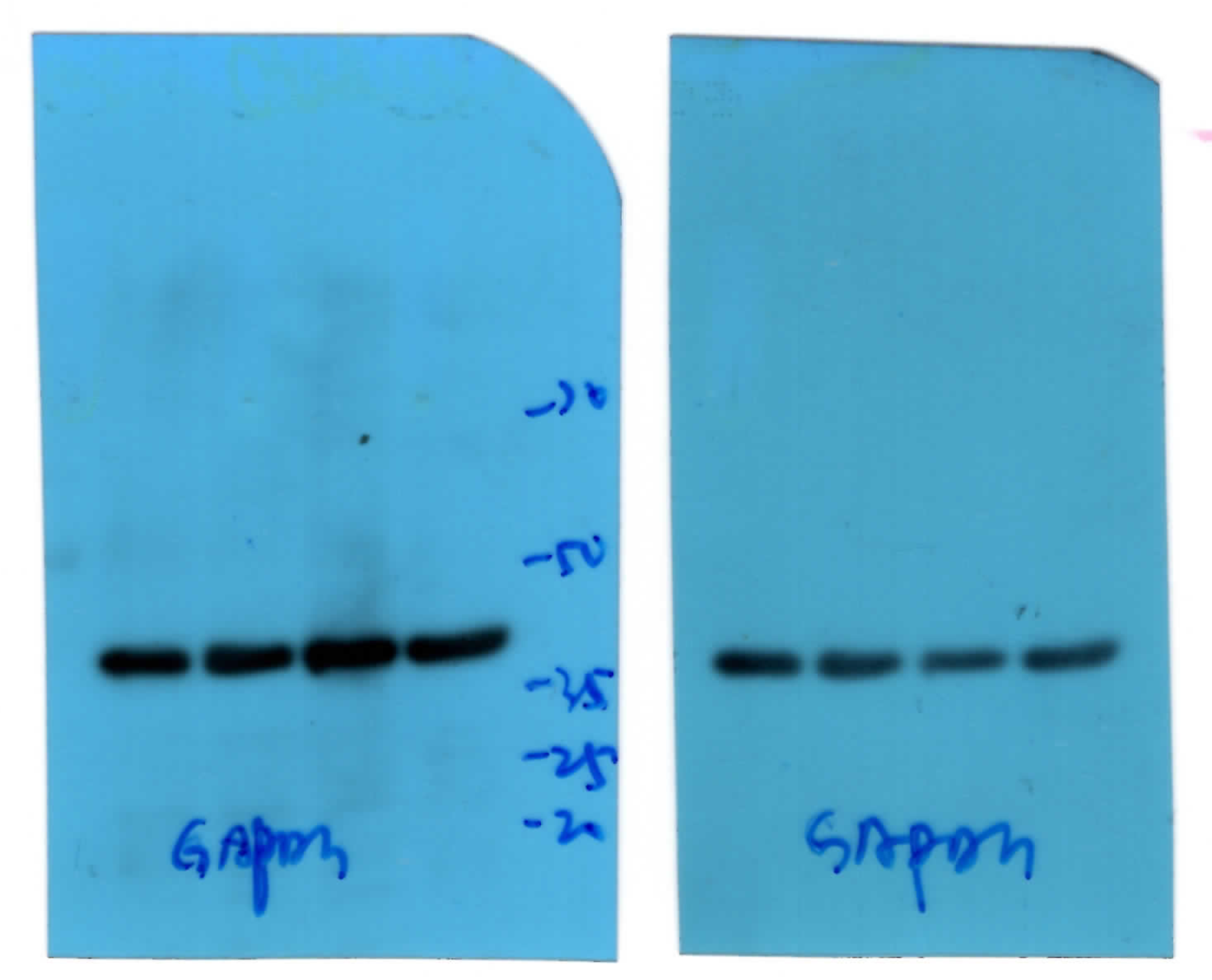


gel figure 3B MG53


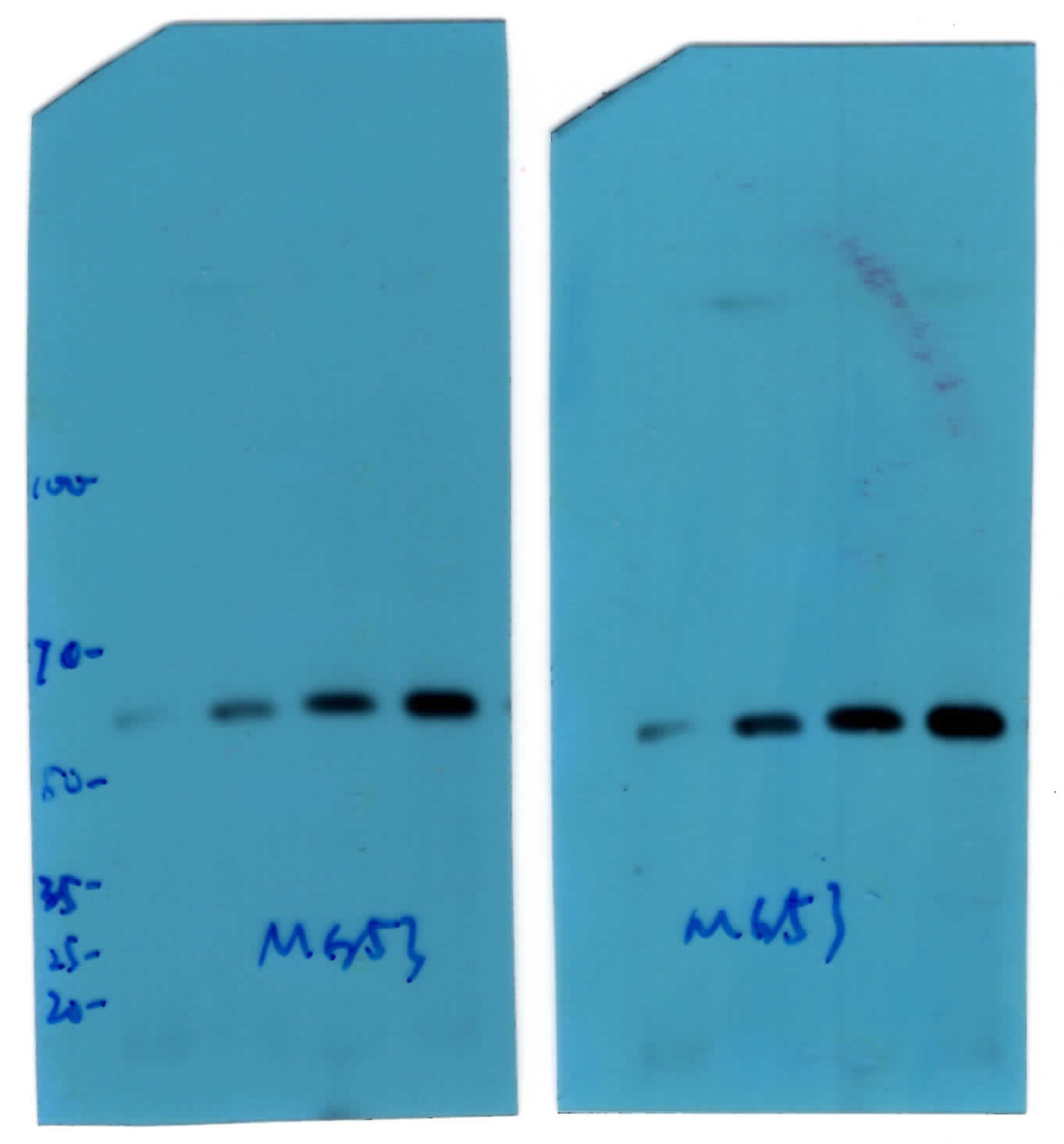


gel figure 3F GAPDH


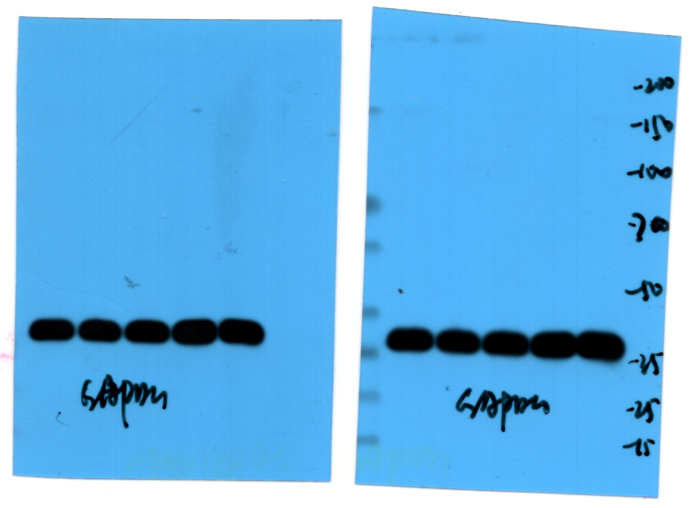


gel figure 3F MG53


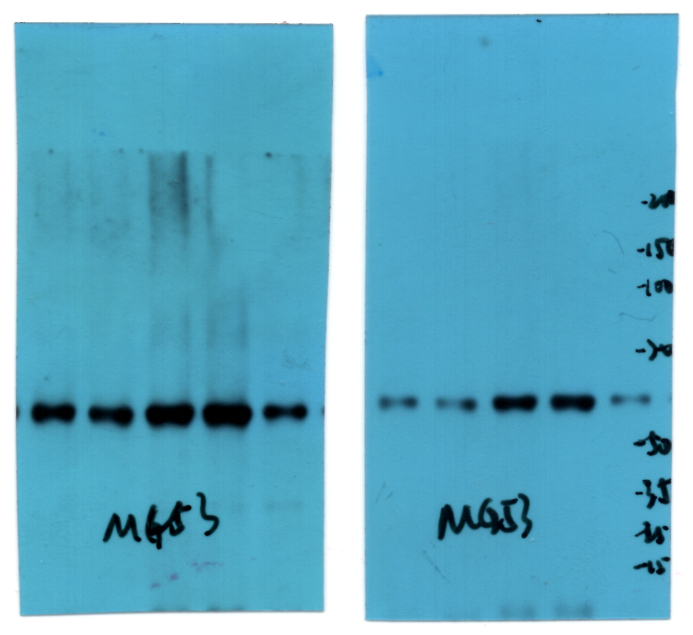


gel supplementary figure 1 CDK2、cleaved caspase-9


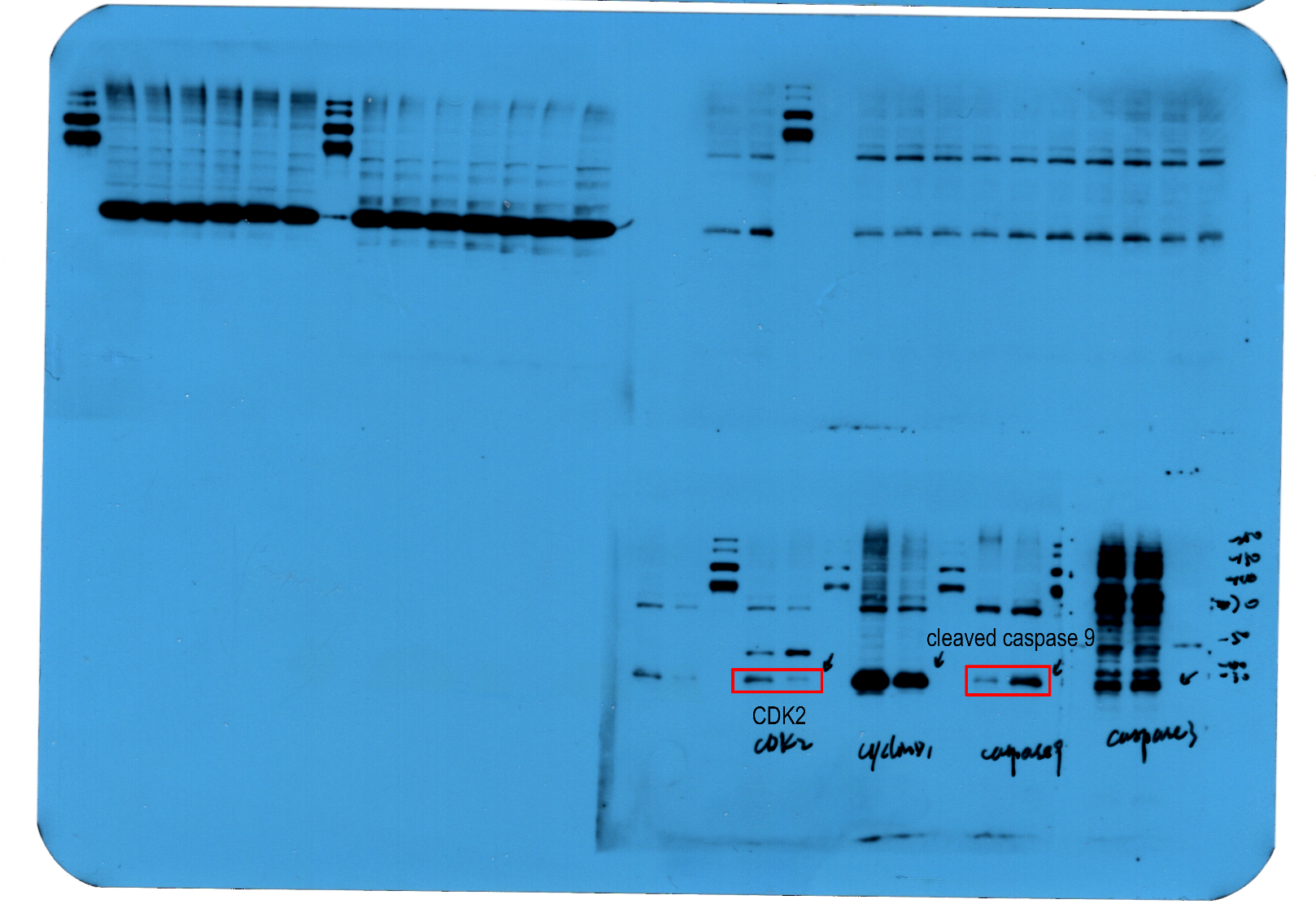


gel supplementary figure 1 cl-caspas3


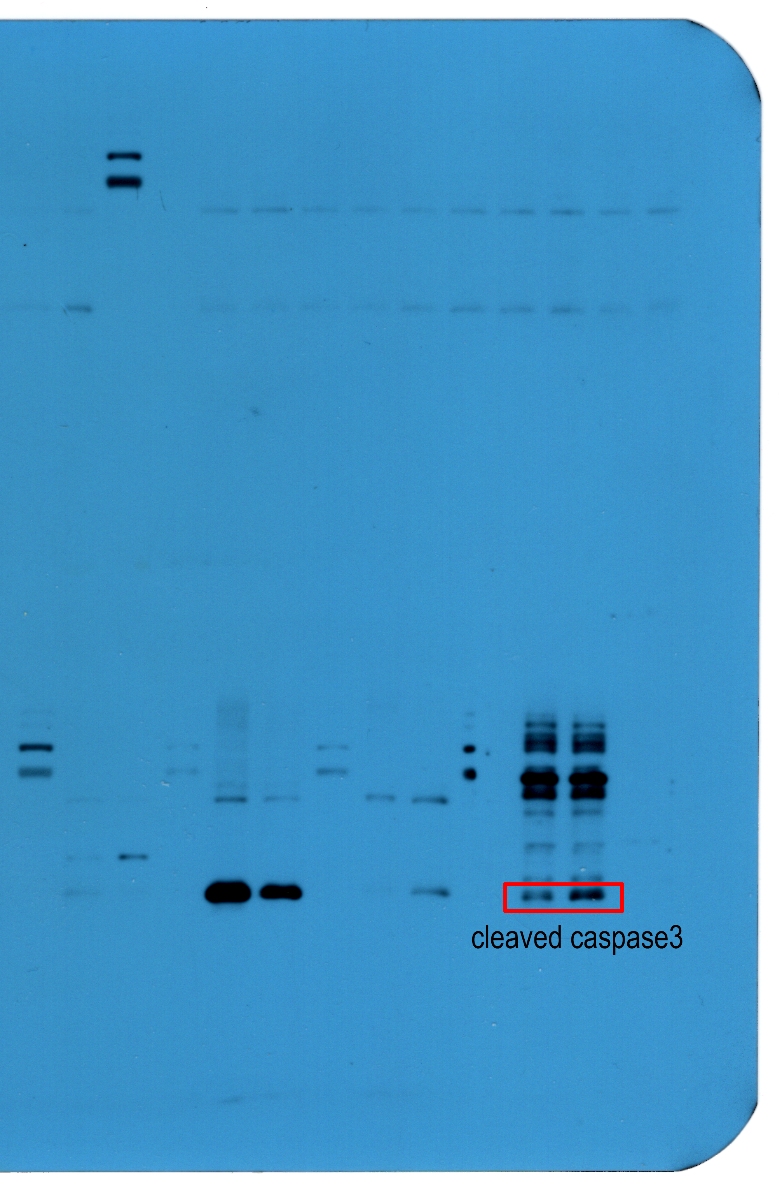


gel supplementary figure 1 cycld1


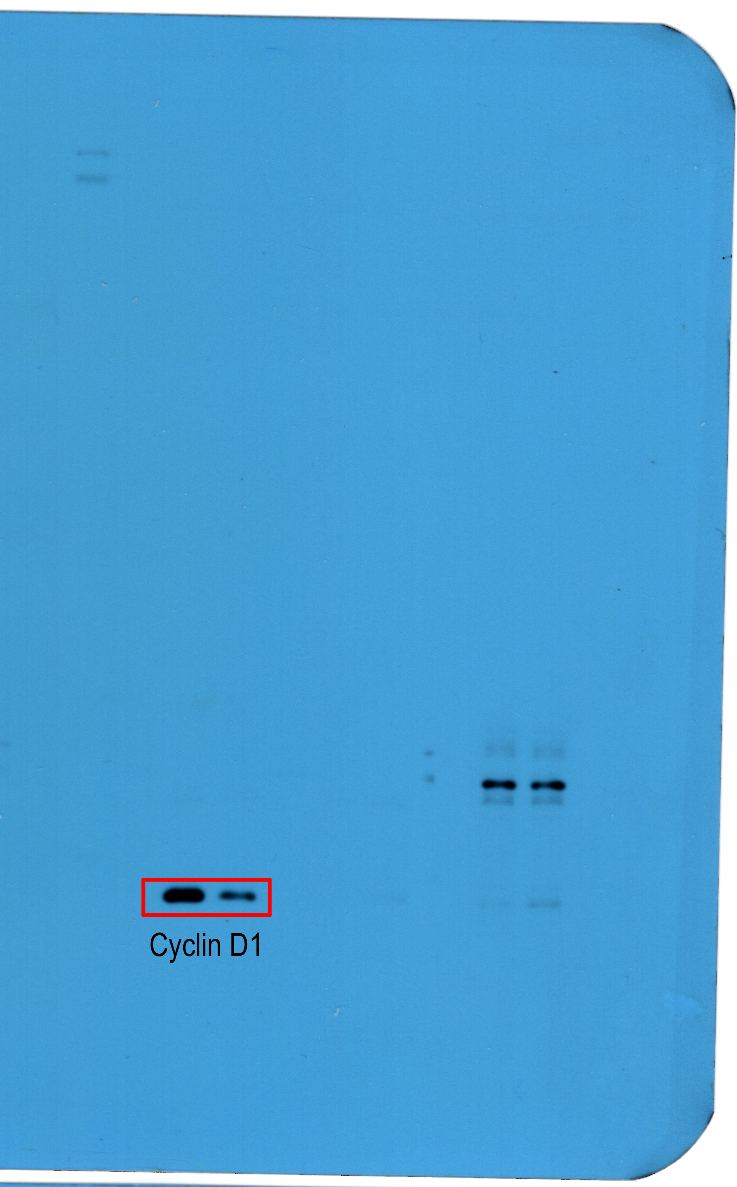


gel supplementary figure 1 GAPDH、P65、p-P65


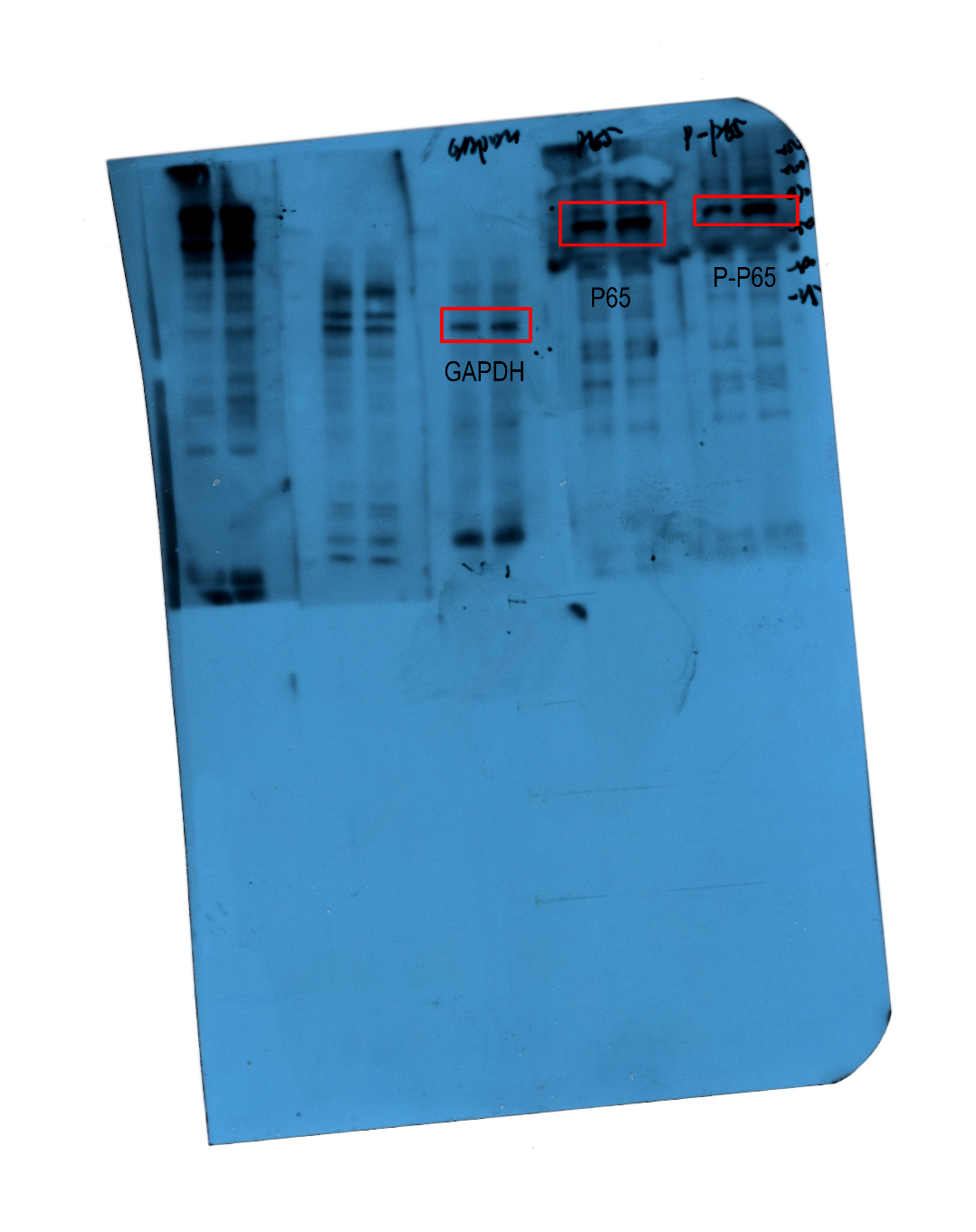

Supplement: Supplementary file 1 — Additional file 1: One-way ANOVA with the Tukey’s post-hoc test of cytokine levels, western blotting analysis of MG53 knockdown on the expression levels of signaling components supplementar, and raw data of western blotting. [file 12872_2022_2458_MOESM1_ESM.docx]
